# The Role of Advanced Practitioners in Optimizing Clinical Management and Support of Patients With Polycythemia Vera

**Published:** 2018-01-01

**Authors:** Sandra Kurtin, Lindsey Lyle

**Affiliations:** The University of Arizona Cancer Center, Tucson, Arizona; and University of Colorado Anschutz Medical Campus, Aurora, Colorado

## Abstract

**CASE STUDY**

Mr. M, a 65-year-old male, presented to his primary care physician with progressive fatigue, difficulty sleeping, and daily headaches for the past 3 weeks. His headaches were not associated with visual disturbances, cognitive deficits, or nausea/vomiting, and he had no history of migraines. He had a history of hypertension and hyperlipidemia, did not smoke, rarely drank alcohol, and had no recent illnesses or hospitalizations. His previous physical examination and laboratory studies 2 years ago were normal. The current physical examination revealed a plethoric yet well-appearing, well-nourished male in no acute distress. His lungs were clear to auscultation bilaterally without wheezes, rales, or rhonchi. He had a regular heart rate and rhythm without murmur. His abdomen was soft, without tenderness, distension, or palpable hepatosplenomegaly. Examination of the extremities was negative for edema. Distal pulses and sensation in the hands and feet were intact and equal bilaterally. Cranial nerves II to XII were deemed intact, and no gross focal deficits were observed. Complete blood count (CBC) revealed a slightly elevated white blood cell (WBC) count (14.6 × 10^9^/L [normal range, 3.9–10.7 × 10^9^/L; Wians, 2015]), erythrocytosis (red blood cell [RBC] count, 6.5 × 1012/L [normal range, 4.2–5.9 × 1012/L; Wians, 2015], hemoglobin, 19 g/dL [normal range, 14–17 g/dL; Wians, 2015], and hematocrit, 54.3% [normal range, 41%–51%; Wians, 2015]), thrombocytosis (platelet count, 500 × 109/L [normal range, 150–350 × 10^9^/L; Wians, 2015]), and microcytosis (mean cell volume [MCV], 75 fL [80–100 fL; Wians, 2015]), which combined were cause for referral to a hematology/oncology clinic. During his hematology/oncology evaluation, Mr. M described "never feeling rested" and being unable to sleep with uncertain snoring habits. He was experiencing itching during hot showers yet did not have rashes and had not recently introduced a new soap. He had no family history of blood disorders and no personal history of blood clots. The second CBC and laboratory tests confirmed erythrocytosis (RBC count, 6.5 × 1012/L; hemoglobin, 18.9 g/dL; hematocrit, 54%) and microcytosis (MCV, 75 fL). Serum iron (22 μg/dL [normal range, 60–160 μg/dL]) and ferritin (5 ng/mL [normal range, 15–200 ng/mL]) were suggestive of iron deficiency, serum erythropoietin was 8 mU/mL (normal range, 4.0–18.5 mU/mL), and a Janus kinase 2 (JAK2) mutation analysis was positive for JAK2V617F. Platelet count remained 500 × 109/L and WBC count was 10.2 × 10^9^/L.

There are an estimated 100,000 patients with polycythemia vera (PV) in the United States (Mehta, Wang, Iqbal, & Mesa, 2014). Because there is no curative medical treatment option (Vannucchi, 2014), these patients require disease management for the remainder of their lives. Patients with PV have an increased risk of mortality (Hultcrantz et al., 2012), often because of cardiovascular or thromboembolic events (Tefferi et al., 2013), and experience burdensome signs and symptoms (Emanuel et al., 2012; Tefferi et al., 2013). Advanced practitioners (APs), including nurse practitioners, PAs, and pharmacists, play key roles in managing these patients. Knowledge of current diagnostic criteria and management strategies is critical to prolonging survival and improving quality of life (QOL).

## PATHOBIOLOGY AND DIAGNOSIS

Polycythemia vera is a myeloproliferative neoplasm (MPN) that is distinguished by erythrocytosis (Tefferi & Vardiman, 2008). Most patients with PV have activating mutations in *JAK2*, including *JAK2*V617F (95%–97%) and *JAK2* exon 12 mutations (2%–4%; Pardanani, Lasho, Finke, Hanson, & Tefferi, 2007; Passamonti et al., 2010). Under normal conditions, JAK2 is a key regulator of hematopoiesis (Quintás-Cardama, Kantarjian, Cortes, & Verstovsek, 2011); however, constitutive activation is associated with PV disease features, including excessive hematopoiesis and splenomegaly (Quintás-Cardama et al., 2011).

**Case Study Continued**

Mr. M was diagnosed with PV per the 2008 World Health Organization (WHO) guidelines (Table 1; Tefferi, Thiele, Vannucchi, & Barbui, 2014; Tefferi & Vardiman, 2008). He presented with the two major criteria (i.e., erythrocytosis and the *JAK2*V617F mutation) and one minor criterion (i.e., low or lower end of normal serum erythropoietin levels).

**Table 1:**
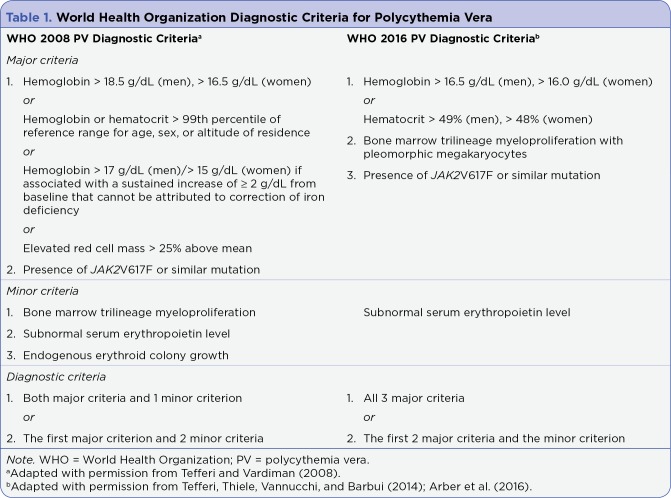
World Health Organization Diagnostic Criteria for Polycythemia Vera

Although the WHO guidelines are the standard for diagnosing PV, they may not aid in identifying patients with masked PV, a condition characterized by *JAK2* mutations and PV-consistent bone marrow morphology, despite subthreshold hemoglobin levels (Barbui et al., 2014). Consequently, the 2016 WHO guidelines include lower thresholds for hemoglobin levels and inclusion of bone marrow results in the major criteria (Table 1).

## NATURAL HISTORY AND DISEASE BURDEN

Mr. M presented with fatigue, headaches, pruritus, and difficulty sleeping. Patients with PV may also present with a thromboembolic event or other disease-related signs, including a palpable spleen (Tefferi et al., 2013). However, many patients are diagnosed asymptomatically based on routine blood work (Passamonti, 2012). Iron deficiency may result from the expanding erythrocyte population with a lower-than-normal erythropoietin level, and may mask the diagnosis of PV (Kambali & Taj, 2016). As the disease progresses, patients are at risk for cardiovascular, thromboembolic, and hemorrhagic events and may develop solid malignancies (Marchioli et al., 2005; Tefferi et al., 2013). Polycythemia vera may also transform to myelofibrosis (MF), which is diagnosed by 2008 International Working Group for Myelofibrosis Research and Treatment (IWG-MRT) criteria (Table 2; Barosi et al., 2008), and/or transform to acute myeloid leukemia (AML), which is identifiable by ≥ 20% blasts in the peripheral blood or bone marrow as well as by extramedullary tumoral blast proliferation (myeloid sarcoma) per 2008 WHO criteria (Vardiman et al., 2009). Cardiovascular or thromboembolic events, solid malignancies, and disease transformation to MF or AML are the leading contributors to an increased mortality risk for patients with PV compared with the age- and sex-matched general population (Hultcrantz et al., 2012; Marchioli et al., 2005; Tefferi et al., 2013). Splenomegaly-associated symptoms, including early satiety, and abdominal discomfort are not uncommon in patients with PV (Emanuel et al., 2012). Based on patient-reported experiences, PV-related symptoms reduce QOL and may hinder activities of daily living and work productivity (Mesa et al., 2016a).

**Table 2:**
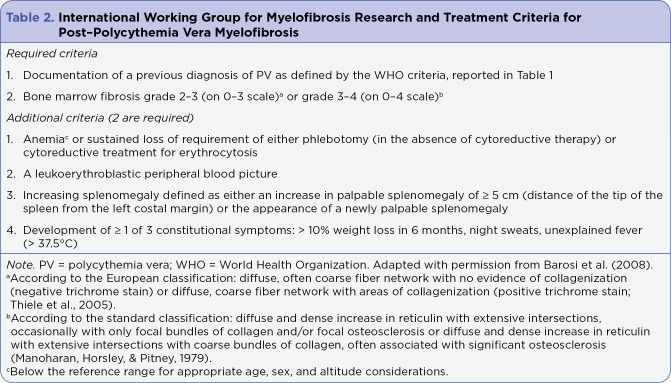
International Working Group for Myelofibrosis Research and Treatment Criteria for Post–Polycythemia Vera Myelofibrosis

## MANAGEMENT

**Treatment Goals**

The treatment goals for PV are to reduce thromboembolic and hemorrhagic risk, to manage disease-related symptoms, and to minimize the risk of fibrotic or leukemic transformation (Barbui et al., 2011). Patients with a history of thromboembolic events and those aged ≥ 60 years have increased thrombotic risk (Barbui et al., 2014; Vannucchi, 2014). In addition, the Cytoreductive Therapy in PV (CYTOPV) trial demonstrated the importance of controlling hematocrit and possibly WBC count. Maintaining a hematocrit < 45% was associated with a fourfold reduced risk of death from cardiovascular or thrombotic events compared with hematocrit maintenance between 45% and 55% (Marchioli et al., 2013). Similarly, WBC count < 7 × 10^9^/L was associated with a fourfold reduced risk of major thrombosis compared with ≥ 11 × 10^9^/L (Barbui et al., 2015). In our case, the patient presented at 65 years of age with a hematocrit of 54%, indicating high risk for thromboembolic events.

**Figure 1:**
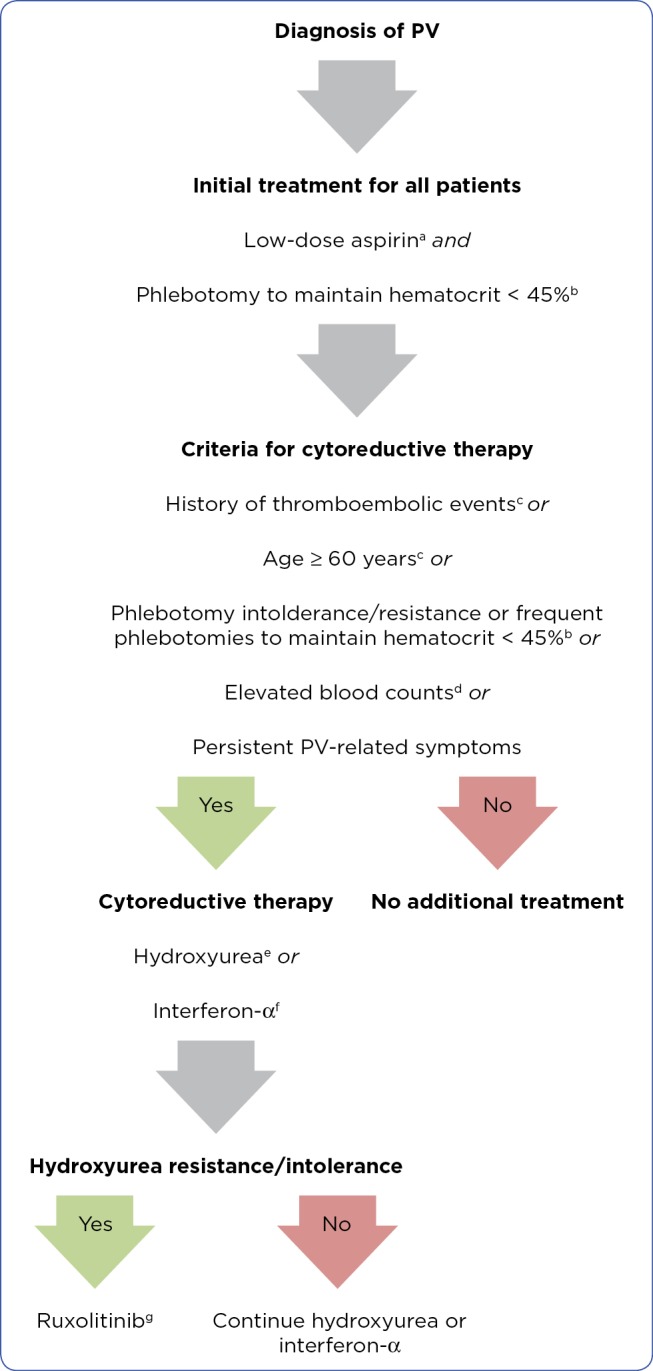
Treatment flow chart for patients with polycythemia vera. PV = polycythemia vera. ^a^Low dose aspirin is contraindicated in patients with extreme thrombosis (platelet count > 1,000 × 109/L; Landolfi et al., 2004). ^b^Marchioli et al. (2013). ^c^Marchioli et al. (2005); Tefferi et al. (2013). ^d^Barbui et al. (2015); Marchioli et al. (2013). ^e^Kiladjian, Chevret, Dosquet, Chomienne, and Rain (2011). ^f^Quintás-Cardama et al. (2009); Sacchi et al. (1994); Silver (2006). ^g^Vannucchi et al. (2015).

**Figure 2:**
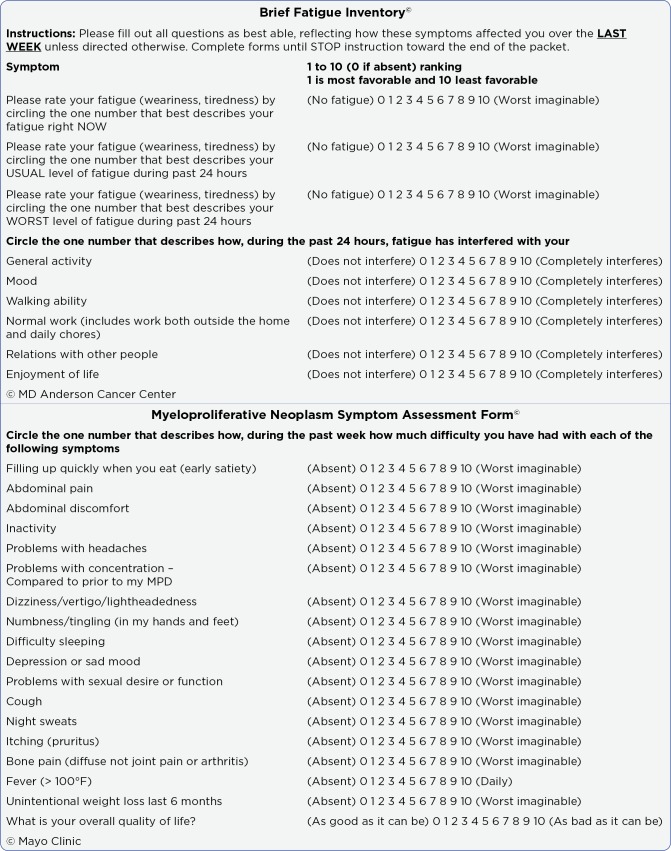
Myeloproliferative Neoplasm Symptom Assessment Form. MPD × myeloproliferative disorder. Reproduced with permission from Scherber et al. (2011)

It is important that APs develop an evidence-based treatment plan (Figure 1). Patients should be monitored routinely for changes in hematocrit and blood count (Barbui et al., 2011), with the frequency dictated by the patient’s disease severity and risk of complications and/or progression. Those with stable blood count may require follow-up every 3 months, whereas some patients, including those with a recent diagnosis or change in treatment, may require weekly monitoring to achieve hematocrit control and appropriate dosing of cytoreductive therapy. Symptom burden should be regularly assessed with an objective instrument such as the MPN Symptom Assessment Form (Figure 2; Barbui et al., 2011; Scherber et al., 2011). Finally, it is important to address comorbidities and history of cardiovascular, thromboembolic, and hemorrhagic events. An emphasis should be placed on achieving optimal control of conventional cardiovascular risk factors (i.e., elevated cholesterol, current smoking status, diabetes mellitus, and high systolic blood pressure; Goff et al., 2014) and monitoring for signs of disease transformation to MF (Table 2) (Barosi et al., 2008) or AML (Vardiman et al., 2009).

**Case Study Continued**

Following diagnosis, Mr. M received weekly phlebotomies until hematocrit was < 45% and started low-dose aspirin (81 mg/d), as is recommended for patients with PV (Landolfi et al., 2004; Marchioli et al., 2013) unless specific contraindications to low-dose aspirin exist, such as extreme thrombocytosis (platelet count > 1,000 × 10^9^/L), at which point acquired von Willebrand syndrome should be ruled out due to risk of bleeding (Tefferi & Barbui, 2015). Because his age was > 60 years, the patient was considered high risk for thrombosis, and therefore started cytoreductive therapy with hydroxyurea.

**Traditional Treatment Options**

Best practices should be used for phlebotomy, including temporary cessation of the phlebotomy or administration of intravenous fluids for hypotension or other phlebotomy-related symptoms (e.g., acute illness, dizziness, dehydration; Parker, Deel, & Arner, 2004). Patients who are high risk (≥ 60 years or with a history of thromboembolic events) should be started on cytoreductive therapy. Additionally, patients with poor disease control with aspirin and phlebotomy alone (continually elevated blood count and/or persistent signs or symptoms) may benefit from the addition of cytoreductive treatment (Barbui et al., 2011) with hydroxyurea (Fruchtman et al., 1997; Kiladjian, Chevret, Dosquet, Chomienne, & Rain, 2011) or interferon-α (Hasselbalch, 2011).

Hydroxyurea is often recommended for first-line cytoreductive therapy (Vannucchi, 2014); however, approximately 1 in 4 patients become resistant or intolerant (Alvarez-Lárran et al., 2012) per European LeukemiaNet (ELN) criteria (Table 3). Hydroxyurea resistance is associated with a 5.6-fold increased risk of death compared with hydroxyurea responders (Alvarez-Lárran et al., 2012). Approaches to the treatment of PV—including indications, common adverse events, and clinical implications—are provided in Table 4.

**Table 3:**
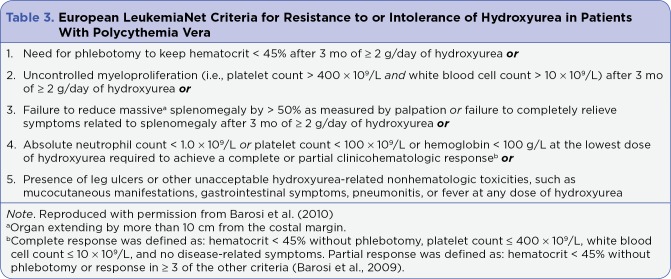
European LeukemiaNet Criteria for Resistance to or Intolerance of Hydroxyurea in Patients With Polycythemia Vera

**Table 4:**
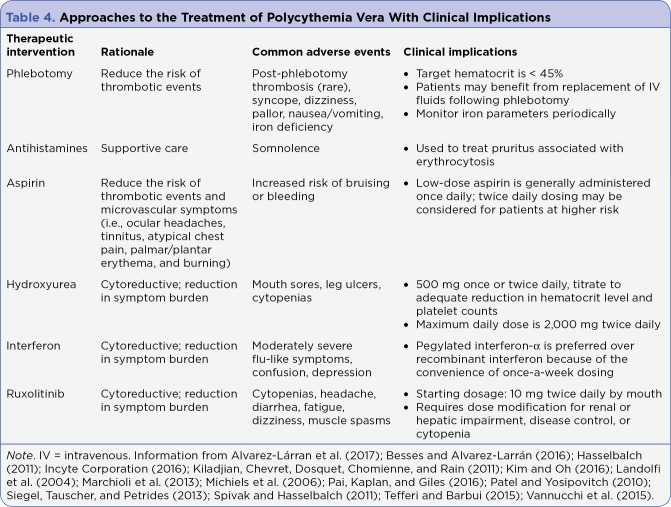
Approaches to the Treatment of Polycythemia Vera With Clinical Implications

**Case Study Continued**

Mr. M’s disease was successfully managed for a few years. In regular follow-up, the WBC count increased over the course of a year to 20 × 10^9^/L, and the patient developed progressive fatigue, pruritus, and night sweats, despite administering hydroxyurea at the maximum tolerated dose, indicating hydroxyurea resistance.

**Treatment Options for Patients With PV Resistant to or Intolerant of Hydroxyurea**

Ruxolitinib (Jakafi [US] or Jakavi [non-US]) is the only agent approved by the US Food and Drug Administration (FDA) for patients with PV who have had an inadequate response to or are intolerant of hydroxyurea (Incyte Corporation, 2016) and by the European Medicines Agency (EMA) for adult patients with PV who are resistant to or intolerant of hydroxyurea (EMA, 2015). Although interferon-α is an effective treatment option for some patients, it is not indicated by the FDA or EMA for patients with PV. Long-term treatment with interferon-α may be challenging for some patients because of its inconvenience as an injectable medication and treatment-related adverse events, which may include chills, depression, diarrhea, fatigue, fever, headache, musculoskeletal pain, myalgia, nausea, and weight loss (Hasselbalch, 2011). For these reasons, we did not consider interferon-α for Mr. M.

Regulatory approval of the oral JAK1/JAK2 inhibitor ruxolitinib (Quintás-Cardama et al., 2010) was based on the randomized, open-label, multicenter phase III RESPONSE trial, which evaluated ruxolitinib vs. best available therapy (BAT; i.e., hydroxyurea, interferon-α, anagrelide, immunomodulators, pipobroman, and observation alone) in patients with PV who required phlebotomy to control hematocrit, had splenomegaly, and were resistant to or intolerant of hydroxyurea (Vannucchi et al., 2015). Ruxolitinib was superior to BAT for hematocrit control without phlebotomy, reduction in enlarged spleen size, and normalization of blood count (Vannucchi et al., 2015). In addition, treatment with ruxolitinib may reduce the severity of PV-related symptoms and improve QOL based on patient-reported outcomes (Mesa et al., 2016b; Vannucchi et al., 2015).

Adverse events with ruxolitinib were primarily grade 1 or 2, with a lower grade 3 or 4 adverse event rate (28.8 per 100 patient-years of exposure) compared with BAT (44.0 per 100 patient-years of exposure; Vannucchi et al., 2015). Among patients treated with ruxolitinib, MF and AML transformation rates were consistent with published rates for high-risk patients with PV (Finazzi et al., 2005; Passamonti et al., 2004; Vannucchi et al., 2015). Herpes zoster infections were all grade 1 or 2 and only occurred with ruxolitinib (6.4%); no patients discontinued ruxolitinib because of herpes zoster (Vannucchi et al., 2015). Nonmelanoma skin cancer occurred in more patients in the ruxolitinib arm (3.6%) compared with the BAT arm (1.8%); however, all patients in the ruxolitinib arm had a history of nonmelanoma skin cancer or precancerous lesions (Vannucchi et al., 2015). No patients died while receiving randomized treatment; two patients died for reasons that were considered unrelated to ruxolitinib treatment after crossing over to ruxolitinib from BAT.

**Case Study Continued**

Because of hydroxyurea resistance, Mr. M was evaluated for treatment with ruxolitinib. Hepatic, renal, and platelet function were noted to be within normal limits. Review of the medication profile did not reveal any potential drug-drug interactions. Therefore, he was started on ruxolitinib 10 mg twice daily. After 3 months of treatment, Mr. M maintained hematocrit control and had improvements in symptoms and leukocytosis.

Ruxolitinib exposure may be affected by hepatic and renal impairment (Chen et al., 2013), as well as concomitant treatment with a cytochrome P450 3A4 inhibitor (Shi et al., 2012), and dosing should be modified as appropriate (Incyte Corporation, 2016). Mr. M was informed about the risks for herpes zoster infections and nonmelanoma skin cancer and continues to be monitored accordingly.

## ASPECTS OF INDIVIDUALIZED PATIENT MANAGEMENT

Advanced practitioners play a critical role in managing patients with PV. Regular patient contact enables the AP to closely monitor laboratory values to optimize medical management and to educate patients about potential disease symptoms, adverse events related to current and/or alternative treatment options, and lifestyle or treatment modifications to achieve improvements (Raedler, 2014). When dispensing medication, pharmacists are provided an opportunity to inform patients about the specifics of treatment delivery and dosing as well as potential drug-drug interactions for those patients receiving concomitant medications. Finally, APs can facilitate improved patient compliance and adherence by placing reminder phone calls to patients, offering direct-to-patient deliveries, and coordinating with health plans and patient assistance programs to facilitate payment.

## DISCUSSION

Patients with PV require long-term management to prolong survival and improve QOL. Familiarity with the 2008 WHO diagnostic criteria (Tefferi & Vardiman, 2008), as well as possible exceptions for masked PV (Barbui et al., 2014), will allow the AP to quickly and accurately identify patients with PV. Although nearly all patients should initially receive treatment with aspirin (Landolfi et al., 2004) and phlebotomy to achieve a target hematocrit < 45% (Barbui et al., 2011; Marchioli et al., 2013), management should evolve with the natural course of the disease (Barbui et al., 2011). Management decisions should be informed by current evidence, based on both objective measures (e.g., CBC, bone marrow biopsy, manual spleen palpation) and subjective measures (e.g., patient-reported symptoms with comprehensive instruments such as the Myeloproliferative Neoplasm Symptom Assessment Form) to identify patients who will benefit from the addition of cytoreductive treatment such as hydroxyurea (Barbui et al., 2011; Scherber et al., 2011). Knowledge of the ELN criteria for hydroxyurea resistance and intolerance (Barosi et al., 2010) will allow the AP in hematology/oncology to identify high-risk patients who may benefit from a change in therapy, including consideration of ruxolitinib (Vannucchi et al., 2015). Advanced practitioners may also identify high-risk patients in need of an alternate disease management strategy based on the IWG-MRT criteria for fibrotic transformation (Barosi et al., 2008) and the WHO criteria for leukemic transformation (Vardiman et al., 2009). Early diagnosis and evidence-based patient management by APs will promote improved outcomes and better QOL.

**Acknowledgment**

Editorial assistance was provided by Cory Pfeiffenberger, PhD (Complete Healthcare Communications, LLC, an ICON plc company), whose work was funded by Incyte Corporation.
